# Game Analysis, Validation, and Potential Application of EyeToy Play and Play 2 to Upper-Extremity Rehabilitation

**DOI:** 10.1155/2014/279609

**Published:** 2014-12-25

**Authors:** Yu-ping Chen, Michelle Caldwell, Erica Dickerhoof, Anastasia Hall, Bryan Odakura, Kimberly Morelli, Hsin-Chen Fanchiang

**Affiliations:** ^1^Department of Physical Therapy, Georgia State University, P.O. Box 4019, Atlanta, GA 30302-4019, USA; ^2^Center for Pediatric Locomotion Sciences, Georgia State University, Atlanta, GA 30302-3975, USA

## Abstract

*Objective.* To describe and analyze the potential use of games in the commercially available EyeToy Play and EyeToy Play 2 on required/targeted training skills and feedback provided for clinical application. *Methods.* A summary table including all games was created. Two movement experts naïve to the software validated required/targeted training skills and feedback for 10 randomly selected games. Ten healthy school-aged children played to further validate the required/targeted training skills. *Results.* All but two (muscular and cardiovascular endurance) had excellent agreement in required/targeted training skills, and there was 100% agreement on feedback. Children's performance in required/targeted training skills (number of unilateral reaches and bilateral reaches, speed, muscular endurance, and cardiovascular endurance) significantly differed between games (*P* < .05). *Conclusion.* EyeToy Play games could be used to train children's arm function. However, a careful evaluation of the games is needed since performance might not be consistent between players and therapists' interpretation.

## 1. Introduction

Recently, virtual reality (VR) has been explored as a training device for improving arm function in adults following stroke [1–5] and for children with cerebral palsy (CP) [6–8]. VR is a computer technology that creates an artificial but highly realistic graphical context and populates it with dynamic objects that allow users to interact with that context [9–12]. The process enables the creation of an exercise environment in which participants, either patients with stroke or children with CP, can practice their arm movements intensively and receive visual and auditory feedback. There are several inexpensive, commercial VR gaming systems available now (e.g., PlayStation 2 with EyeToy Camera, Nintendo Wii systems, and Microsoft Kinect system), which increase the accessibility of utilizing VR systems for rehabilitation purposes like training arm function. Consequently, researchers have begun to investigate the effects of these commercially available games on the improvement of arm function in patients with stroke or children with CP [1, 2, 5–8]. The research has shown the potential for using the games to improve some aspects of arm function.

There are challenges in applying commercially available VR systems that were designed for recreation to do rehabilitation [13]. Deutsch et al. [13] noted that some interfaces might require adaptation (e.g., the Wii remote controller requires good hand control) and the level of difficulty of the games might not be suitable for some players, especially those with impaired arm function. Moreover, the skills required to play games might vary, which makes game selection difficult.

Deutsch et al. [13] created a detailed “game analysis table” to describe the games listed in Wii Sports and Wii Fit (Nintendo of America, Inc., Redmond, Washington), including game-related features (description, scoring, and progression), equipment used, length of game, feedback provided (knowledge of results or knowledge of performance), and impairments that can potentially benefit from the training (balance, coordination, endurance, strength, and upper-extremity control). They validated their game analyses by rating the agreement of two experienced physical therapists who were naïve to the games on feedback and impairment type. The researchers reported 100% agreement between raters on ratings for impairment type and between 50% to 100% agreement on feedback provided.

In this study, we analyzed and validated games in the SONY PlayStation 2 EyeToy Play and EyeToy Play 2 (Sony Computer Entertainment American LLC, San Meteo, CA). EyeToy Play was selected because this system uses a USB camera as the method to capture players' motions, so that the players can see themselves as they are immersed in the virtual world. This software has been used in several studies to train arm movements in patients with stroke or children with CP and has been found effective [1, 2, 5–8]. Moreover, at $150 for a new system, the selected unit is at the low end of the price range for commercially available video consoles. It thus has the greatest potential to be widely used in clinics and in children's homes. The purpose of this paper is to (1) provide a detailed summary table describing all the games in EyeToy Play and EyeToy Play 2 and (2) analyze and validate the specific games in EyeToy Play and EyeToy Play 2 for their potential to train for upper-extremity function in children. Finally, specific recommendations for upper-extremity function in children are also discussed.

## 2. Materials and Methods

This study included two phases: Phase I—creating a game summary table and Phase II—validating the items in the summary table using two movement experts (Phase II-1) and 10 typically developing children (Phase II-2).

### 2.1. Phase I: Game Analysis and Game Summary Table Creation

The first author (YC), who was experienced in using EyeToy Play and EyeToy Play 2 games to train children with cerebral palsy, created the items needed for inclusion in the game analysis table. These items were similar to Deutsch et al. [13] and included game features (e.g., goal of the game as listed by the software brochure, object to interact with), required/targeted training skills (e.g., unilateral reaching, bilateral reaching), feedback provided (e.g., knowledge of results), and special notes/comments (e.g., “game rules are unclear”). All items in the required/targeted training skills and feedback categories were defined based on motor learning and rehabilitation references (see Table 1 for definitions) [14–18]. Next, the first author and five physical therapy students played all the games as many times as needed to become conversant with them (range 5–8 times) and worked together to summarize each game in EyeToy Play and EyeToy Play 2 using the items listed earlier to describe the game, required/targeted training skills, feedback provided, and special comments.  Table 2 provides an example of the “Beat Freak” game from EyeToy Play.

### 2.2. Phase II: Validation Process of Game Summary Table

#### 2.2.1. Validation by Movement Experts

To determine agreement on the required/targeted training skills and feedback sections of the game summary table, a physical therapist rater (KM) with more than 10 years of clinical experience in physical therapy and a movement scientist (HF) with a background in motor learning, biomechanics, and motion analysis evaluated 10 games randomly. The games were selected by putting all the relevant names from the game list into a bag and then randomly picking 10, similar to the method used by Deutsch et al. [13]. Six games from the EyeToy Play (Beat Freak, Kung Fu, Rocket Rumble, Slap Stream, Soccer Craze, and Wishi Washi) and four games from the EyeToy Play 2 (Bubble Pop, Goal Attack, Table Tennis, and Kung 2) were selected.

The two raters were naïve EyeToy players and seldom played video games. A short instruction session was conducted to go over the definitions of each item in the required/targeted training skills and feedback sections. The raters took turns playing and observing each other playing the game, and they were allowed as much time as they wanted for this phase. They then made ratings independently of each other after playing the game. Ratings were based on each rater's own playing experience as well as their observations of the other person. All items were rated either a “yes” or a “no.” The raters also commented on whether to recommend the game as part of a therapeutic program for children who need to train their upper-extremity function. Percentage of agreement between the raters was then calculated. After the rating process was completed, a discussion session was held between the two raters and the first author in order to arrive at consensus on any inconsistent ratings. Changes were made in the game summary table if the consensus was different from the summary table (2 games on muscular endurance and 2 games on cardiovascular endurance were changed after discussion).

#### 2.2.2. Validation by Testing Healthy Typically Developing Children

We further validated the required/targeted training skills section in our game summary table by having healthy, typically developing children play EyeToy Play and EyeToy Play 2 games. In this paper, we presented only unilateral reaching, bilateral reaching, speed, muscular endurance, and cardiovascular endurance as these items did not reach 100% agreement during the validation process by two movement experts (see Section 3 for details). We intentionally used children with typical development in this validation process because we needed to establish our reasonable expectations for children's regular performance in the commercial games before using a group of children with clinical diagnosis.

*Participants*. Ten children aged 6 to 12 years participated in this validation process (mean age: 8.20 ± 1.69 years old, 6 females 4 males). All participants were recruited from flyers or by word of mouth and were reported by their parents to be free of any neurological or orthopedic diseases and to have typical physical and cognitive development. Parents or legal guardians of the children signed an informed consent form prior to testing, and oral assents were obtained from the children.

*Apparatus*. The game console used was PlayStation 2 with EyeToy camera. The image was projected to a large screen on the wall (size: 264 cm × 220 cm) to create better visibility for the children to interact with the virtual objects in the game. Nine games from the Validation Phase 1 were tested with these children, including Beat Freak, Bubble Pop, Goal Attack, Kung Fu, Rocket Rumble, Slap Stream, Soccer Craze, Table Tennis, and Wishi Washi. One game (Kung 2) from the previous list was not used because (1) this game was very similar to the original Kung Fu, which might have left the children feeling bored and uninterested, and (2) the agreement on this game between the two movement experts was already excellent. The order of games to be played was randomized (using a table of random numbers) by a student who was not aware of the study purpose and naïve to these games.

Participants' heart rate was measured using a Puma Children's Heart Rate monitoring system. A digital camera (Apple, iPhone 5, at 30 Hz) was placed about 15 degrees from the child's midline in front of the child to record the whole play sessions. 

*Procedures*. All the testing took place in a university classroom. After assent and consent were obtained from children and their parent or legal guardians, participants placed the heart rate monitor strap around their chest and were asked to sit quietly for 5 minutes in order to measure their resting heart rate. Then the participants played nine EyeToy games in random order. One researcher was seated behind the participant to obtain the heart rate measurement from the heart rate monitor. For each game, the participant practiced the game for about 30 seconds before the recording started. The participant was instructed to play the game for 3 minutes, and heart rate was recorded at initiation and at 1-minute intervals. Rarely, if the game was accidentally interrupted before 3 minutes, a researcher reactivated the game immediately. Participants took a 5-minute break after finishing each game and listened to the researcher explaining the rules for the next game. This break allowed participants' heart rate to return to its regular level. The parent was present throughout the testing period.

*Data Reduction*. Video data were exported to a computer and coded by the first author using Windows Movie Maker. The number of unilateral reaches, bilateral reaches, and total reaches was coded from the video using slow-motion and a frame-by-frame mode. Unilateral reaches were defined as the unilateral extension of one arm towards the location of a virtual object [19]. While one arm was moving, the other arm was held still, regardless of position (e.g., held at shoulder height or at the side of the body). Bilateral reaches were defined as simultaneous performance of both arm movements. To constitute a bilateral reach, the two arms did not need to move in the same direction, but both arms had to be moving. Our definitions were consistent with those of Deutsch et al. [13] and Corbetta and Thelen [20]. Unilateral and bilateral reaches were coded starting around 30 seconds after play started and coding continued for 1 minute. We did not code the complete trial or use the complete dataset to avoid the possible influence of fatigue on reaching frequency and also to avoid having a familiarization period, even though the participants had practiced before data collection. Since children could have a different number of reaches in the period of data collection, we converted the number of unilateral and bilateral reaches into percentages to normalize individual differences. The number of reaches per minute was used to represent speed. Reaches per minute were derived by combining the number of unilateral reaches and bilateral reaches within the coded minute. The higher number of reaches per minute is produced, the faster movement is required (i.e., faster speed).

We used the total number of reaches in the 3-minute interval as an indicator of muscular endurance since the more the reaches done by players, the greater the chance that the players would experience fatigue of the arm. If their muscular endurance was not good, they might do fewer reaches during the 3-minute session.

We also determined whether playing the games required cardiovascular endurance by measuring players' heart rate at initiation and at 1-minute intervals. Four different heart-rate related variables were computed to represent the potential usefulness of each game for training cardiovascular endurance: maximal heart rate change, maximal heart rate, average heart rate, and percentage of heart rate reserve ([maximal heart rate during the game − resting heart rate]/[(208 − age ∗ 0.7) − resting heart rate]) [21–23].

*Analysis*. For this observational study, we used descriptive statistics to report reaches and heart rate change in each game. The percentages of unilateral and bilateral reaches with each game, the number of reaches per minute, total number of reaches, and maximal heart rate change, maximal heart rate, average heart rate, and percentage of heart rate reserve with each game were compared between games using repeated analysis of variance (ANOVA), with games as the repeated factor. A preplanned paired *t* test was used to determine where the differences were once the repeated ANOVA reached significance. All the analyses were conducted using SPSS 18.0.

## 3. Results

### 3.1. Game Summary Table

The completed game summary table for the EyeToy Play and EyeToy Play 2 games is in Table 3, which includes game related features, required/targeted training skills, feedback provided, and recommendations. A summary of the number of games in EyeToy Play and EyeToy Play 2 that could potentially train the required/targeted skills is given in Table 4.

There are 12 games each in the EyeToy Play and EyeToy Play 2 software. Our analyses showed that 11 games in EyeToy Play and 10 games in EyeToy Play 2 enabled players to practice unilateral reaching; all the games in EyeToy Play and 8 games in Play 2 enabled practice of bilateral reaching. Most of the games targeted speed, accuracy, eye-hand coordination, and strength. Muscular endurance was targeted in 9 games each in EyeToy Play and EyeToy Play 2. Only 3 games in EyeToy Play and 1 game in EyeToy Play 2 targeted cardiovascular endurance, but this finding might be due to the difficulty in monitoring a player's heart rate without the proper apparatus (i.e., a heart rate monitor). All the games provided “Knowledge of Results” (KR) feedback, and all games but one provided “Knowledge of Performance” (KP) feedback.

Seven games from EyeToy Play and 9 games from EyeToy Play 2 are recommended to children who need to train upper-extremity function (see Table 3). Games were not recommended for children for the following reasons: four games (Plate Spinner, Mirror Game, UFO Juggler, and Monkey Bar) were not recommended because of difficult or confusing game rules, one game (Ghost Eliminator) was not recommended because of the scary scene, one game (Air Guitar) had no real reaching movements, and one game (Home Run) has a small and blurred display of the player on the screen. Among the recommended games, all but one (Goal Attack) can be used to train unilateral reaching, and all but two (Table Tennis and Secret Agent) can be used to train bilateral reaching. All of the recommended games target speed and strength and can provide KR and KP feedback. Four games in EyeToy Play and 8 games in EyeToy Play 2 require some cognition involvement; five games in EyeToy Play and all games in EyeToy Play 2 target accuracy during play.

### 3.2. Validation of Game Summary Table—by Two Independent Raters

Agreement between the two experts on items in the required/targeted training skills section ranged between 70% and 100%; agreement on items in the feedback category was 100%. The lowest agreement scores were on endurance-related items (70% and 80% for muscular and cardiovascular endurance, resp.). Agreement between raters on each game ranged between 83% and 100%, with the lowest agreement on Soccer Craze (83%). Percent agreement between the raters is shown in Table 5.

### 3.3. Validation of Game Summary Table—by Testing 10 Children

*Unilateral and Bilateral Reaching*. All these games produced both unilateral and bilateral reaches. However, there were statistically significant differences between games (*F*(8,32) = 4.565, *P* = .001). Children who played Slap Stream used mainly unilateral reaching, rather than bilateral reaching. Kung Fu, Rocket Rumble, Beat Freak, Table Tennis, Bubble Pop, and Soccer Craze also elicited more unilateral than bilateral reaching (see Table 6). Children used equal amount of unilateral and bilateral reaching when they played Wishi Washi, and they mainly used bilateral reaching when playing Goal Attack. These differences were statistically significant (*P* < .05).

*Speed*. Number of arm movements ranged from 15.17 to 79.10 per minutes and differed statistically different between games (*F*(8,32) = 3.416, *P* = .006). Children did more movements per minute when playing WishiWashi and Rocket Rumble and fewer when playing Goal Attack. 

*Muscular Endurance*. Muscular endurance was operationalized as the total number of reaching movements in the 3-minute playing interval. Wishi Washi produced the greatest number of reaches, followed by Bubble Pop, Rocket Rumble, Kung Fu, Soccer Craze, and Beat Freak. Goal Attack produced the smallest number of reaches. Table Tennis and Slap Stream also tended to produce fewer reaches than other games. The difference between games for number of reaches was statistically significant (*F*(8,64) = 3.55, *P* = .002).

*Cardiovascular Endurance*. Cardiovascular endurance was operationalized using maximal heart rate change, maximal heart rate, average heart rate, and percentage of heart rate reserve as indicators. All of these variables indicated similar trends: Soccer Craze, Kung Fu, and Wishi Washi elicited the largest maximal heart rate change, maximal heart rate, average heart rate, and percentage of heart rate reserve, followed by Beat Freak, Rocket Rumble, and Table Tennis. Slap Stream, Goal Attack, and Bubble Pop had the smallest heart rate change, maximal heart rate, average heart rate, and percentage of heart rate reserve.

## 4. Discussion

The primary goals of this study were to provide summary of the games in EyeToy Play and EyeToy Play 2 and to validate the game table for future use by clinicians. Seven games from the EyeToy Play and 9 games from the EyeToy Play2 are recommended for children who need to train upper-extremity function. Almost all of the 16 games can be used to train unilateral and bilateral reaching movements and provide proper feedback. Some games, however, require more cognitive involvement than others, and some games can be specifically used to train for accuracy or for speed of reaching movements. The detailed game analysis table can also help clinicians select the games they recommend to their clients for use in training reaching movements.

Agreement by movement experts on the required/targeted training skills rating ranged from 70% to 100%. As expected, the two items with the lowest agreement were related to endurance (muscular endurance and cardiovascular endurance). Muscular endurance was defined as “the ability of muscle to sustain forces repeatedly or to generate forces over a period of time,” but even though we suggested the use of total arm movements during the 3-minute period, this was hard to operationalize because the strategies used to play the games varied slightly between players. For example, when playing the game of Soccer Craze, the two movement experts (i.e., the two raters) in our study used different strategies to complete the game: Rater 1 used quick and short arm movements to keep the soccer ball in the air, while Rater 2 used a different strategy, moving the arm slowly but more precisely. Both strategies worked since both raters were able to play the game for at least 3 minutes. Cardiovascular endurance was also difficult to rate since it is difficult to be measured directly. The rest of the required/targeted training skills were highly consistent between the two raters, showing excellent validity of the game analysis table.

Unlike the rating scheme for feedback (KR or KP) developed by Deutsch et al. [13], the two raters agreed 100% on whether the individual games they rated could provide KR and/or KP. One possible explanation would be that we used a dichotomous variable (yes/no) to rate feedback, rather than a Likert scale, which narrowed the range of responses and thus increased the likelihood of agreement. Another possible explanation is that the EyeToy games had a USB camera to capture the player's movements on screen, providing one-to-one corresponding movements, which made the rating of feedback for KP very easy since the player could see his/her movement on the screen.

Our study further validated those games which did not have a perfect agreement by 10 healthy, typically developing children playing the games. We videotaped the children playing the games in a random order and used a heart rate monitor to measure their heart rates. We coded their reaches and counted their total reaching movements during the 3-minute game. Interestingly, although all 9 games elicited unilateral and bilateral reaching, some games produced more unilateral reaches than others (e.g., Slap Stream) and some games elicited more bilateral reaches than others (e.g., Goal Attack). The number of arm movements per minute was used to represent the speed in the required/targeted training skills. Wishi Washi and Rocket Rumble were the two games required fast speed, whereas Goal Attack did not. We used the count of total arm movements during the entire 3-minute session to represent muscular endurance and four heart rate related measures to represent cardiovascular endurance. Wishi Washi, Bubble Pop, and Rocket Rumble produced more arm movements than the other games, indicating that these games might be used for training more muscular endurance if players can finish the game. Interestingly, all 4 heart rate related measures indicated similar trends among the 9 games: Soccer Craze, Kung Fu, and Wishi Washi increased heart rate more than Slap Stream, Goal Attack, and Bubble Pop.

Generally, the ratings between the movement experts and children's performance were quite consistent in unilateral reaching, bilateral reaching, speed, and muscular endurance. Only one game (Goal Attack) differed between the experts and children's performance in speed, which was operationally defined as number of arm movements per minute. The experts rated all games as requiring/targeting speed; however, the children's performance showed that Goal Attack generated just 15 arm movements per minute (in other words, it was relatively slow). Using the standard reported by Lythgo et al. [24, 25], a typical school-aged child doing a daily activity like walking can generate about 30 arm movements per minute, yet Goal Attack produced a slower frequency of arm movements, which was different from the experts' rating. Unilateral reaching, bilateral reaching, and muscular endurance were consistent between the experts' ratings and the children's performance.

Cardiovascular endurance was the item showing the most inconsistency between the experts' rating and children's performance. In the expert's rating, only Wishi Washi required/targeted cardiovascular endurance. However, from the children's performance, all games on average reached at least 33% of their heart rate reserve. It is worth noting that 4 games (Kung Fu, Rocket Rumble, Soccer Craze, and Wishi Washi) exceeded 40% of heart rate reserve, which is the recommendation of the American College of Sport Medicine for aerobic training [18]. This suggests that these games would have the potential to train children's cardiovascular endurance. This inconsistency when rating cardiovascular endurance suggests that therapists might be based on their own experience and expertise to make the exercise prescription to children which may not be accurate because children's movement strategies may differ from adults. If this is true, then therapists would need to observe the children in action in order to make correct recommendations.

There are some limitations in this study. The sample size used in this study was only 10, though the children performed quite consistently among themselves. Future studies should include a larger sample and should also include children with need to train arm function (e.g., cerebral palsy) since their responses may not be the same as those of children with typical development. In addition, the heart rate monitor used in the current study could not store heart rate data; therefore, the actual amount by which heart rate exceeded the training zone (40% of heart rate reserve) could not be calculated. Future studies should include a more sensitive heart rate monitor to examine the effect of VR games on cardiovascular endurance. Also, our definition of “speed” was based on number of arm movements per minute which might not be the best definition, as we did not directly measure the speed of the arm movements. A sensitive motion analysis system and eye tracker may be needed to examine the speed and accuracy of participants' movements and even eye-hand coordination.

## 5. Conclusion

Our study provides a detailed summary table for the games in Sony PlayStation 2 EyeToy Play and EyeToy Play2. Moreover, although these games are not designed specifically for children who need to train their arm function, our research shows that some of the games studied could be useful therapeutic tools to improve their reaching abilities. For example, if the goal is to target unilateral reaching, Slap Stream might be a good game to train for that. If the goal is to train muscular endurance, Wishi Washi, Bubble Pop, and Rocket Rumble might be the best choices. If cardiovascular endurance is the training goal, Soccer Craze, Kung Fu, and Wishi Washi might be the best games. The advancement of new technology has promise to move treatment forward at a low cost; however, a careful evaluation of the games is needed since performance might not be consistent between players and therapists' interpretation.

## Figures and Tables

**Table 1:** Definitions of required/targeted training skills and feedback provided.

	Conceptual definition used in Phases I and II-1 validation by two movement experts	Operational definition used in Phase II-2 validation by testing healthy children
*Required*/*targeted training skills *		
Unilateral reaching	Movements of the upper extremities that use 1 hand or arm.	Number of upper-extremity movements using 1 hand between 30 seconds after play started and 1 minute and 30 seconds.
Bilateral reaching	Movements of the upper extremities that use 2 hands or arms. Bilateral reaching can be symmetrical (both arms perform the same joint motions) or alternative (e.g., one arm is extending while the other is flexing).	Number of upper-extremity movements using both hands between 30 seconds after play started and 1 minute and 30 seconds.
Speed	The game requires the player to reach faster, since faster is better.	Number of arm movements per minute
Cognition	This game requires some cognitive abilities. For example, someone with intellectual disabilities may not understand the game rules and may not be able to play the game.	†
Accuracy	The game requires some precision.	†
Muscular endurance	The ability of muscle to sustain forces repeatedly or to generate forces over a period of time. Muscular endurance refers to the body's ability to continue using muscular strength and endure repeated contractions for an extended period of time. Usually, if the game requires the player to constantly repeat the same arm movements over time, it requires muscular endurance.	Total number of arm movements performed in the 3-minute interval
Cardiovascular endurance	The ability of the body to sustain prolonged rhythmical exercise and perform work and participate in an activity over time. Cardiovascular endurance is the power, strength, or ability of the heart to supply enough oxygen to muscles during a physical activity for a prolonged period of time. It essentially indicates how strong one's heart is and can potentially add years to one's life. This can be measured by heart rate change.	Four heart rate related indicators: maximal heart rate change, maximal heart rate, average heart rate, and percentage of heart rate reserve = (maximal heart rate during the game—resting heart rate)/[(208—age ∗ 0.7) —resting heart rate]
Eye-hand coordination	The coordinated control of eye movement with hand movement. The ability to guide the movements of the hand with the eyes.	†
Strength	Muscle force exerted by a group of muscles to overcome a resistance in a specific set of circumstances.	†
*Feedback provided *		
Knowledge of results	Information about the outcome of the action Individual action: information about the outcome of each action (e.g., a banging sound after hitting an object). Whole game: information about the outcome after playing the whole game (e.g., the total score of the game, number of opponents being hit)	†
Knowledge of performance	Information about the pattern of action, for example, the player can see his/her movement while performing the task.	†

†: reach 100% agreement during the validation process by two movement experts in Phase II-1. It did not include Phase II-2 validation.

**Table 2:** Example of a complete game analysis table, using “Beat Freak” from EyeToy Play.

	Beat freak
(I) Game features	
Goal of the game	Follow the CDs to hit the speakers to play the music.
Context	Reach to strike the speakers in the four corners of the screen just as the CD reaches them. There are a few cartoon characters at the bottom of the screen dancing and cheering for the player; however, they may impede the player's view of their movements when hitting the speakers at the two lower corners.
Objects to interact with	CDs
Object location	From the center to one of the 4 speakers located in the 4 corners.
Appearance of the object	About 1.3-1.4 seconds
Total duration of the game	3 minutes
Number of objects to interact with at once	1–4
Disturbing effects	None
Avoiding objects/effects	None
Speed of object appearance	10 CDs/the first 20 seconds
Method to advance to different levels within the same game	None
Method to end the game	Time is up or miss 3 CDs
(II) Required skills	
Unilateral reaching	Yes
Bilateral reaching	Yes (for both symmetrical or asymmetrical)
Speed	Yes
Cognition	Yes
Accuracy	Yes
Muscular endurance	Yes
Cardiovascular endurance	No
Eye-hand coordination	Yes
Strength	Yes
(III) Feedback provided	
Knowledge of results: individual action	Yes: auditory (when hitting the speaker) and visual (speaker lights up)
Knowledge of results: whole game	Yes: score
Knowledge of performance	Yes: the player can see his/her movement
(IV) Special notes	Increase in speed and randomness throughout the game

**Table 3:** Game analysis table for the games in EyeToy Play and EyeToy Play 2.

Game	Goal	Context	Unilateral	Bilateral	Speed	Cognition	Accuracy	Muscular endurance	CV endurance	Eye-hand coordination	Strength	KR individual	KR-whole game	KP	Recommendation
EyeToy Play															
Beat Freak	Follow the CDs to hit the speakers to play the music.	Reach to strike the speakers in the four corners of the screen just as the CD reaches them.	Y	Y	Y	Y	Y	Y	N	Y	Y	Auditory and visual	Score	Player's mvts	Y
Slap Stream	Hit the mice, but not the bunny girls.	Four clouds located at 4 corners with mouse or bunny appearing randomly.	Y	Y	Y	Y	Y	Y	N	Y	Y	Auditory and visual	Score	Player's mvts	Y
Wishi Washi	Clean as many windows as possible before the time runs out.	A window with soap all over needs to be cleaned using any upper-extremity movements. When soap is cleaned, the player will be seen.	Y	Y	Y	N	N	Y	Y	N	Y	Visual	Number of windows	Trajectory of hand mvts	Y
Kung Foo	Knock off the creatures and break the boards during bonus game.	Two towers located at the sides with creatures coming out from upper or lower windows or from the ground.	Y	Y	Y	N	Y	Y	N	Y	Y	Auditory and visual	Score	Player's mvts	Y
Soccer Craze	Keep the soccer ball(s) from hitting the ground as well as trying to hit the “bad guys” in the windows.	Two buildings located at each side with “bad guys” randomly appearing from one of the windows to interrupt hitting the soccer ball. The soccer ball is falling from the sky and the player needs to keep it up.	Y	Y	Y	Y	Y	Y	N	Y	Y	Visual	Score	Player's mvts	Y
Rocket Rumble	Catch the rocket to create a firework show.	Rockets appear from the bottom of the screen. The player needs to touch the rockets with the same color and then push handle at either side to create a firework show.	Y	Y	Y	Y	Y	Y	N	Y	Y	Auditory and visual	Score	Player's mvts	Y
Boxing Chump	Try to knock out the opponent in the boxing match.	Player must sit at an angle to the screen and use as many arm movements as possible to knock out the opponent and avoid being hit.	Y	Y	Y	N	N	Y	Y	Y	Y	Auditory	Score and energy bar	Player's mvts	Y
Plate Spinner	Spin the plates and try to keep the monkeys from knocking the plates over.	Four plate spinners located at each side with two higher and two lower. The player needs to constantly move the spinners to prevent the plates from falling.	N	Y	Y	Y	N	Y	Y	Y	Y	Visual	Score	Player's mvts	N
Mirror Game	Hit the green balls but avoid the red ones.	Four balls located at 4 corners of the screen. The player needs to hit the green balls but not the red ones. The screen will flip right/left and upside down (mirrored images) to make this game very difficult	Y	Y	Y	Y	Y	N	N	Y	Y	Auditory and visual	Score	Player's mvts	N
Ghost Eliminator	Wave the player's hand over the ghosts and bats to make them disappear.	Ghosts and bats appear from different locations of the screen. The scene displays a cemetery and a dark castle at night, which may be scary to kids.	Y	Y	Y	N	Y	N	N	Y	Y	Visual	Score	Player's mvts	N
Disco Stars	Imitate the dance moves of the disco girl on stage.	A disco star dances on the stage, and the player needs to copy her moves and rhythm. The star will point to 1 or 2 of the 5 lights on the top of the screen and the player needs to imitate the moves and tempo.	Y	Y	N	Y	Y	N	N	Y	Y	Visual (written comment)	Score	Player's mvts	N
UFO Juggler	Spin the UFOs so they can elevate and fly off to safety.	UFOs randomly appear from the bottom of the screen. The player needs to constantly wave to keep spinning the UFOs, but too much spinning will also cause UFOs to blow up.	Y^*^	Y^*^	N	Y	Y	Y	N	Y	Y	Visual	Score	Player's mvts	N

EyeToy Play 2															
Bubble Pop	Reach with one or both arms to pop all the blue bubbles and avoid popping the red bubbles before the time runs out.	The locations and proportions of the blue and red bubbles vary at different levels. The more advanced the level is, the harder it is to pop all the blue bubbles as they may require very precise aiming movements.	Y	Y	Y	Y	Y	Y	N	Y	Y	Auditory and visual	Score	Player's mvts	Y
Goal Attack	Use both arms to block the soccer ball to the side or above the player's head.	The player needs to successfully block the soccer ball from entering the net during practice trial (a ball machine) or from the opponent team member.	Y	Y	Y	Y	Y	N	N	Y	Y	Auditory and visual	Score	Player's mvts	Y
Table Tennis	Use the player's hands as paddles to hit the ping-pong balls.	Different opponents to compete and bonus round (e.g., to break the glass bottles, to squeeze tankers, etc.)	Y	N	Y	Y	Y	Y	N	Y	Y	Visual	Score	Player's mvts	Y
Kung 2	Punch all small ninjas appearing from different parts of the screen.	Small ninjas may appear from any location of the screen. The player needs to hit the ninjas and ninjas' weapons to avoid losing “lives.”	Y	Y	Y	Y	Y	Y	N	Y	Y	Auditory and visual	Score and energy bar	Player's mvts	Y
DIY	Successfully complete common household tasks and chores.	Eight household tasks randomly appear: grab saw, leaky pipes, stack bricks, wood chipper, demolish wall, tiles, and cutting logs, and hammer the nails.	Y^†^	Y^†^	Y	Y	Y	Y	N	Y	Y	Auditory and visual	Score	Player's mvts	Y
Air Guitar	The player pretends to play a guitar, constantly strumming and cued to “grasp” the cord on the neck of the guitar at regular intervals.	A guitar appears on the screen and the player pretends to play. A cue is offered and the player needs to “catch” the falling “cues” and then strum the guitar in order to play music.	N	N	N	N	N	N	N	N	N	Auditory and visual	Score and music	Only player's fingers appear on screen. No KP	N
Secret Agent	Reach in all directions to capture the assigned objects that are falling from the top of the screen or present at any locations of the screen.	Different scenarios with different assignments: (1) prison cell—grab 6 hacksaws to avoid the cameras or be perfectly still to avoid detection; (2) rooftop—collect 8 ropes to avoid the search light or be perfectly still to avoid detection; (3) courtyard—steal 8 keys and avoid the search light.	Y	N	Y	Y	Y	Y	N	Y	Y	Visual	Score and words (“mission complete”)	Player's mvts	Y
Drumming	Strike certain drums as the game cues them to	Six drums on the lower half of the screen: 3 at each side. A red note cue flies from the center to the drum and the player needs to strike the drum accordingly.	Y	Y	Y	Y	Y	Y	N	Y	Y	Auditory and visual	Score and music	Player's mvts	Y
Home Run	To hit the baseball to make it a home run. An indicator shows in front of the player to help the player determine the timing to hit the ball.	The player would be positioned as a hitter to hit the baseball. There are crowds cheering, and there is a virtual pitcher that throws the baseball. The virtual distance the ball flies will be displayed like a real baseball game.	Y	Y	Y	Y	Y	Y	N	Y	Y	Auditory and visual	Score	Player's mvts	Y/N
Mr. Chef	The player makes burgers, milkshakes, and other food.	The player needs to follow the order to grasp the ingredients to make the order within a desired time limit, or to follow the instructions to make the food (e.g., to shake the shaker).	Y	Y	Y	Y	Y	Y	N	Y	Y	Visual	Score	Player's mvts	Y
Knock out	To hit the opponent	Similar to Box Chump in EyeToy Play. The player needs to hit the matched opponent at different angles.	Y	Y	Y	N	Y	Y	Y	Y	Y	Auditory	Score and energy	Player's mvts	Y^‡^
Monkey Bars	Quite difficult to figure out how to play this game.	A tall building with lots of windows. A monkey needs to climb up and down the building using the 4 corner bottoms but not sure how to activate and move the monkey.	Y	?	Y	Y	Y	?	?	Y	Y	Visual	Score	Unclear	N

Abbreviations: CV: cardiovascular; KR: knowledge of results; KP: knowledge of performance; Y: yes; N: no; mvts: movements; Y^*^: For UFO juggler, it is more a waving motion than an arm reaching movement. ^†^For DIY game, it depends on the tasks to involve unilateral and/or bilateral reaching; Y^‡^: although this game can train the child's arm movements, be mindful of violence of this game.

**Table 4:** Summary of number of games that could potentially train the required/targeted skills in EyeToy Play and EyeToy Play 2.

	EyeToy Play	EyeToy Play 2
Unilateral reaching	11 (91.67%)	10 (83.33%)
Bilateral reaching	12 (100%)	8 (66.67%)
Speed	10 (83.33%)	11 (91.67%)
Cognition	8 (66.67%)	10 (83.33%)
Accuracy	9 (75.00%)	11 (91.67%)
Muscular endurance	9 (75.00%)	9 (75.00%)
Cardiovascular endurance	3 (25.00%)	1 (8.33%)
Eye-hand coordination	11 (91.67%)	11 (91.67%)
Strength	12 (100.00%)	11 (91.67%)
Knowledge of results: individual action	12 (100.00%)	12 (100.00%)
Knowledge of results: Whole game	12 (100.00%)	12 (100.00%)
Knowledge of performance	12 (100.00%)	11 (91.67%)
Recommendation	7 (58.33%)	9 (75.00%)

**Table 5:** Percent agreement between two independent raters by game and by item.

By game	Agreement	By item	Agreement
Beat Freak	100.00%	Unilateral reaching	100.00%
Kung Fu	92.31%	Bilateral reaching	90.00%
Rocket Rumble	100.00%	Speed	90.00%
Slap Stream	100.00%	Cognition	100.00%
Soccer Craze	84.62%	Accuracy	100.00%
Wishi Washi	92.31%	Muscular endurance	70.00%
Bubble Pop	92.31%	Cardiovascular endurance	80.00%
Goal Attack	92.31%	Eye-hand coordination	100.00%
Kung 2	100.00%	Strength	100.00%
Table Tennis	92.31%	Feedback provided	100.00%
		Knowledge of results: Individual action	100.00%
		Knowledge of results: Whole game	100.00%
		Knowledge of performance	100.00%
		Recommendation	90.00%

**Table 6:** Quantitative validation of the game analysis table using 10 children with typical development.

Game	Unilateral reaching^*^	Bilateral reaching^*^	Speed^*^ (# arm mvts/min)	Number of total arm mvts^*^	Maximal heart rate change^*^	Maximal heart rate^*^	Average heart rate^*^	Percentage of heart rate reserve^*^
Beat Freak	75.04% (12.42%)	24.96% (12.42%)	51.00 (11.32)	187.88 (97.96)	23.60 (18.33)	132.70 (13.14)	122.40 (12.69)	36.10% (14.51%)
Kung Fu	84.76% (12.75%)	15.24% (12.75%)	49.22 (13.84)	166.89 (82.44)	35.10 (17.52)	147.20 (17.62)	130.13 (16.21)	44.63% (17.87%)
Rocket Rumble	81.42% (23.46%)	18.58% (23.46%)	62.78 (36.21)	253.33 (234.52)	23.00 (18.97)	140.90 (25.43)	128.55 (16.75)	41.24% (19.30%)
Slap Stream	98.78% (2.74%)	1.22% (2.74%)	41.11 (13.73)	125.44 (54.02)	16.30 (9.55)	128.60 (10.52)	120.47 (13.58)	33.18% (10.72%)
Soccer Craze	65.80% (36.88%)	34.20% (36.88%)	35.90 (13.63)	159.89 (64.76)	38.60 (21.14)	154.70 (24.82)	137.78 (17.89)	53.18% (20.47%)
Wishi Washi	54.67% (42.15%)	45.33% (42.15%)	79.10 (32.78)	456.78 (415.93)	33.50 (17.83)	145.60 (17.65)	131.43 (17.23)	46.74% (18.40%)
Bubble Pop	75.52% (12.56%)	24.48% (12.56%)	40.14 (21.42)	335.22 (421.66)	19.10 (7.52)	131.60 (15.79)	121.30 (14.44)	33.84% (11.46%)
Goal Attack	21.00% (22.26%)	79.00% (22.26%)	15.17 (5.85)	67.33 (38.68)	16.00 (9.80)	129.20 (12.02)	121.15 (12.52)	33.30% (11.46%)
Table Tennis	76.59% (34.19%)	23.41% (34.19%)	33.56 (15.37)	125.56 (68.38)	25.20 (16.61)	133.40 (16.22)	121.65 (12.58)	35.17% (16.82%)

^*^ *P* < .05: a statistical significance was found between games; the value in parentheses is the standard deviation; mvts: movements.

## References

[1] Flynn S., Palma P., Bender A. (2007). Feasibility of using the Sony PlayStation 2 gaming platform for an individual poststroke: a case report. *Journal of Neurologic Physical Therapy*.

[2] Neil A., Ens S., Pelletier R., Jarus T., Rand D. (2013). Sony PlayStation EyeToy elicits higher levels of movement than the Nintendo Wii: implications for stroke rehabilitation. *European Journal of Physical and Rehabilitation Medicine*.

[3] Peters D. M., McPherson A. K., Fletcher B., McClenaghan B. A., Fritz S. L. (2013). Counting repetitions: an observational study of video game play in people with chronic poststroke hemiparesis. *The Journal of Neurologic Physical Therapy*.

[4] Rand D., Kizony R., Weiss P. T. L. (2008). The sony playstation II eye toy: low-cost virtual reality for use in rehabilitation. *Journal of Neurologic Physical Therapy*.

[5] Yavuzer G., Senel A., Atay M. B., Stam H. J. (2008). ‘Playstation eyetoy games’ improve upper extremity-related motor functioning in subacute stroke: a randomized controlled clinical trial. *European Journal of Physical and Rehabilitation Medicine*.

[6] Chen Y.-P., Kang L.-J., Chuang T.-Y., Doong J.-L., Lee S.-J., Tsai M.-W., Jeng S.-F., Sung W.-H. (2007). Use of virtual reality to improve upper-extremity control in children with cerebral palsy: a single-subject design. *Physical Therapy*.

[7] Chen Y.-P., Lee S.-Y., Howard A. M. (2014). Effect of virtual reality on upper extremity function in children with cerebral palsy: a meta-analysis. *Pediatric Physical Therapy*.

[8] Jannink M. J. A., van der Wilden G. J., Navis D. W., Visser G., Gussinklo J., Ijzerman M. (2008). A low-cost video game applied for training of upper extremity function in children with cerebral palsy: a pilot study. *Cyberpsychology & Behavior*.

[9] Parsons T. D., Rizzo A. A., Rogers S., York P. (2009). Virtual reality in paediatric rehabilitation: a review. *Developmental Neurorehabilitation*.

[10] Snider L., Majnemer A., Darsaklis V. (2010). Virtual reality as a therapeutic modality for children with cerebral palsy. *Developmental Neurorehabilitation*.

[11] Weiss P. L., Rand D., Katz N., Kizony R. (2004). Video capture virtual reality as a flexible and effective rehabilitation tool. *Journal of NeuroEngineering and Rehabilitation*.

[12] Wilson P. N., Foreman N., Stanton D. (1997). Virtual reality, disability and rehabilitation. *Disability and Rehabilitation*.

[13] Deutsch J. E., Brettler A., Smith C., Welsh J., John R., Guarrera-Bowlby P., Kafri M. (2011). Nintendo Wii sports and Wii fit game analysis, validation, and application to stroke rehabilitation. *Topics in Stroke Rehabilitation*.

[14] Magill R. A. (2010). *Motor Learning and Control: Concepts and Applications*.

[15] Shumway-Cook A., Woollacott M. (2011). *Motor Control: Translating Research into Clinical Practice*.

[16] Schmidt R. A., Lee T. D. (2011). *Motor Control and Learning: A Behavioral Emphasis*.

[17] APTA (2003). *Guide to Physical Therapist Practice*.

[18] ACSM (2013). *ACSM's Guidelines for Exercise Testing and Prescription*.

[19] Fetters L., Todd J. (1987). Quantitative assessment of infant reaching movements. *Journal of Motor Behavior*.

[20] Corbetta D., Thelen E. (1996). The developmental origins of bimanual coordination: a dynamic perspective. *Journal of Experimental Psychology: Human Perception and Performance*.

[21] Machado F. A., Denadai B. S. (2011). Validity of maximum heart rate prediction equations for children and adolescents. *Arquivos Brasileiros de Cardiologia*.

[22] Mahon A. D., Marjerrison A. D., Lee J. D., Woodruff M. E., Hanna L. E. (2010). Evaluating the prediction of maximal heart rate in children and adolescents. *Research Quarterly for Exercise and Sport*.

[23] Robert M., Ballaz L., Hart R., Lemay M. (2013). Exercise intensity levels in children with cerebral palsy while playing with an active video game console. *Physical Therapy*.

[24] Lythgo N., Wilson C., Galea M. (2009). Basic gait and symmetry measures for primary school-aged children and young adults whilst walking barefoot and with shoes. *Gait & Posture*.

[25] Lythgo N., Wilson C., Galea M. (2011). Basic gait and symmetry measures for primary school-aged children and young adults. II: walking at slow, free and fast speed. *Gait & Posture*.

